# Supplementary material for: Gradients in the biophysical properties of neonatal auditory neurons align with synaptic contact position and the intensity coding map of inner hair cells
Source: eLife. 2020 Jul 8;9:e55378. doi: 10.7554/eLife.55378 (PMC7343388; doi:10.7554/eLife.55378)
Supplement: Transparent reporting form [file elife-55378-transrepform.docx]

***eLife’s* transparent reporting form**

We encourage authors to provide detailed information *within their submission* to facilitate the interpretation and replication of experiments. Authors can upload supporting documentation to indicate the use of appropriate reporting guidelines for health-related research (see [EQUATOR Network](http://www.equator-network.org/%20)), life science research (see the [BioSharing Information Resource](https://biosharing.org/)), or the [ARRIVE guidelines](http://www.plosbiology.org/article/info:doi/10.1371/journal.pbio.1000412) for reporting work involving animal research. Where applicable, authors should refer to any relevant reporting standards documents in this form.

If you have any questions, please consult our Journal Policies and/or contact us: [editorial@elifesciences.org](mailto:editorial@elifesciences.org).

**Sample-size estimation**

- You should state whether an appropriate sample size was computed when the study was being designed
- You should state the statistical method of sample size computation and any required assumptions
- If no explicit power analysis was used, you should describe how you decided what sample (replicate) size (number) to use

Please outline where this information can be found within the submission (e.g., sections or figure legends), or explain why this information doesn’t apply to your submission:

These experiments were not amenable to an a priori power analysis to estimate sample size as we did not have prior knowledge about the range of biophysical properties to expect in this preparation. We indicated in the methods that we performed a post-hoc power analysis for each statistical test to determined when sample sizes were sufficient. We collected data from different portions of the ganglion (Figure 1) to ensure that we sampled the full range of contact positions for the innervation pattern previously reported (Kalluri et al 2017). This sampling strategy is reported in the methods. Built into the multiple variable regression analysis was a variable reduction strategy to ensure that the models were not overfit for the sample size. This is described within the methods and reported in Table 2.

**Replicates**

- You should report how often each experiment was performed
- You should include a definition of biological versus technical replication
- The data obtained should be provided and sufficient information should be provided to indicate the number of independent biological and/or technical replicates
- If you encountered any outliers, you should describe how these were handled
- Criteria for exclusion/inclusion of data should be clearly stated
- High-throughput sequence data should be uploaded before submission, with a private link for reviewers provided (these are available from both GEO and ArrayExpress)

Please outline where this information can be found within the submission (e.g., sections or figure legends), or explain why this information doesn’t apply to your submission:

Each experiment was performed independently of each other. Each image in Figure 1D-1E represents individual recordings and single cell labeling. Two to three neurons were labeled in each preparation. In total, we performed independent patch-clamp recordings and immunohistochemistry on 128 cells. This is stated within the methods section of the manuscript.

Exclusion criteria are described in the methods and results. Patch-clamp recordings were excluded if they were made without a gigaohm seal or if we detected significant changes in resting potential. Patch-clamp recordings that did not have traces of transient inward currents (most likely Na+ currents) and had small currents were usually glial cells (verified by labeling) and were excluded. Neurons from animals at age post-natal days 1 through 2 were excluded in the model fitting process as they were few and did not adequately sample contact positions throughout the expected innervation range (Figure 3 and 5).

**Statistical reporting**

- Statistical analysis methods should be described and justified
- Raw data should be presented in figures whenever informative to do so (typically when N per group is less than 10)
- For each experiment, you should identify the statistical tests used, exact values of N, definitions of center, methods of multiple test correction, and dispersion and precision measures (e.g., mean, median, SD, SEM, confidence intervals; and, for the major substantive results, a measure of effect size (e.g., Pearson's r, Cohen's d)
- Report exact p-values wherever possible alongside the summary statistics and 95% confidence intervals. These should be reported for all key questions and not only when the p-value is less than 0.05.

Please outline where this information can be found within the submission (e.g., sections or figure legends), or explain why this information doesn’t apply to your submission:

Our methods for statistical analysis and model fitting are summarized within a dedicated section in the methods called “Statistical Analysis”. The specific statistical test used for individual analyses are stated within the text in the results. In most instances we show raw data as individual points. Precision measures (sample size, means and standard errors) are reported in the text and captions. Effect sizes for correlations are reported as Pearson’s r, embedded in Figures (2,3,4). Exact p-values are printed on the figure and/or caption; values less than 0.001 are reported as p < 0.001. For regression fits we plotted the 95% confidence intervals as well as the line of least squared means with lines (Figure 2,3,4). Significance tests for regressions across categorical variables were performed using a 2-way ANOVAs, the results of which are reported in Tables 3 and 4.

(For large datasets, or papers with a very large number of statistical tests, you may upload a single table file with tests, Ns, etc., with reference to sections in the manuscript.)

**Group allocation**

- Indicate how samples were allocated into experimental groups (in the case of clinical studies, please specify allocation to treatment method); if randomization was used, please also state if restricted randomization was applied
- Indicate if masking was used during group allocation, data collection and/or data analysis

Please outline where this information can be found within the submission (e.g., sections or figure legends), or explain why this information doesn’t apply to your submission:

Data were collected from neonatal rats of both sexes chosen randomly per experiment and over a range of ages from P1 through P16. The criteria for allocating data into age and morphology groups is described within the text relating to each figure.

**Additional data files (“source data”)**

- We encourage you to upload relevant additional data files, such as numerical data that are represented as a graph in a figure, or as a summary table
- Where provided, these should be in the most useful format, and they can be uploaded as “Source data” files linked to a main figure or table
- Include model definition files including the full list of parameters used
- Include code used for data analysis (e.g., R, MatLab)
- Avoid stating that data files are “available upon request”

Please indicate the figures or tables for which source data files have been provided:

We will include a data spreadsheet containing the biophysical quantities and morphological quantification of the recorded and labelled spiral ganglion neurons. These data were used to create the figures and tables in this manuscript.
